# Designing a Web-Based Navigation Tool to Support Access to Youth Mental Health Services: Qualitative Study

**DOI:** 10.2196/48945

**Published:** 2024-01-18

**Authors:** Alison L Calear, Philip J Batterham, Sonia M McCallum, Michelle Banfield, Elizabeth Moore, Natalie Johnson, Alyssa R Morse

**Affiliations:** 1 Centre for Mental Health Research The Australian National University Canberra Australia; 2 ALIVE National Centre for Mental Health Research Translation Melbourne Australia; 3 Office for Mental Health and Wellbeing ACT Health Canberra Australia

**Keywords:** mental health services, youth, navigation tool, mental health, website, user experience, design, service, services, access, accessibility, health care system

## Abstract

**Background:**

Many young people with mental health problems do not readily seek help or receive treatment and support. One way to address low help-seeking behavior is to improve access to information on mental health services and how to navigate the mental health system via a web-based tool. Seeking input from the end users (young people and parents or caregivers) on key features of the tool is imperative to ensure that it is relevant, engaging, and likely to meet their needs and expectations.

**Objective:**

This study aims to investigate young person and parent or caregiver views on the design, content, functioning, and user experience of a web-based mental health navigation tool to support connection to mental health services for children and young people aged up to 25 years.

**Methods:**

A total of 4 online focus groups were conducted: 2 with young people aged 16 years and older (total n=15) and 2 with parents or caregivers (total n=13). Focus groups were structured around a series of guiding questions to explore participants’ views on content, features, user experience, and design of a mental health navigation website. Focus groups were audio recorded with detailed notes taken. In addition, 53 young people aged 16-25 years and 97 parents or caregivers completed an online survey, comprising closed- and open-ended questions; open-ended responses were included with the focus group data in the qualitative analysis. All qualitative data were analyzed using thematic analysis.

**Results:**

A total of 2 topic areas and 7 themes were developed. The first topic area covered the *types of information* needs of young people and parents. Identified themes concerned the scope of the navigation website, as well as the provision of up-to-date and practical information on how to navigate the whole help-seeking process. The second topic area covered *website features* that would be beneficial and included the consideration of the website design; search engines; supported navigation; and forums, reviews, and user accounts.

**Conclusions:**

This study provides important insights into the navigation needs of young people and parents or caregivers in seeking mental health services. Key findings identified through this research have directly informed the development of MindMap, a web-based youth navigation tool providing a searchable database of local services, including a clear description, their location, and potential wait times. The website can be navigated independently or with support.

## Introduction

Globally, mental disorders are common and often emerge during childhood and adolescence [1,2]. The worldwide pooled prevalence of mental disorders in children and adolescents has been reported to be between 12.7% and 13.4% [2,3], while suicide is one of the leading causes of mortality in this age group [4]. Timely access to appropriate services and supports for mental disorders and suicidal distress can mitigate the persistence of poorer health, academic, and social outcomes into adulthood [5].

Research suggests, however, that many young people do not readily seek or receive treatment or support for psychological distress, suicidal ideation, or suicidal behavior [3,6,7]. A recent systematic review and meta-analysis of the prevalence of mental disorders in children and adolescents in high-income countries found that only 44.2% of young people with mental disorders received any services for their conditions [3]. Another study conducted in Australia reported slightly higher rates of service use among children and adolescents, with 56% accessing services for emotional and behavioral problems [7]. However, Sawyer et al [8] reported that only 12% of Australian young people aged 6-17 years with a mental health condition received what was considered adequate treatment.

Several possible drivers of low help-seeking behavior among young people, parents, and other caregivers have been identified. A recent review found that limited mental health knowledge, embarrassment and perceived social stigma, a lack of perceived confidentiality and trust in mental health providers, financial costs, logistical barriers, and limited availability of services were common barriers to accessing professional help for mental health problems among young people [5]. Similar barriers were identified among parents seeking treatment for mental health problems in their children and adolescents, with a lack of knowledge of where or how to seek help and a limited understanding of the mental health system identified as key barriers to service use [9].

One way to address some of these barriers may be to provide young people and parents or caregivers with a navigation tool they could use to identify available services and traverse often complex and disconnected mental health systems. Similar to other high-income countries, there are multiple entry points into the mental health care system in Australia, but access is often dependent on the individual knowing about them. As such, being able to access all service options in the same place would overcome service knowledge barriers and save users’ time trying to identify what is available in their area. Providing such tools online through an interactive website would also make this information more accessible, as adolescents and parents report seeking mental health information online [10-13] and a website would allow greater reach and timely updating of service information. In a survey assessing parents’ help-seeking for their adolescent’s mental health, over 75% of participants indicated that they would use the internet to find information about services [14]. In developing such a website, it is imperative that young people and parents or caregivers are involved, as there is clear evidence in the literature that the involvement of end users is essential to ensuring uptake and that the website meets the needs and expectations of users [15,16].

This paper reports the outcomes of focus groups and online surveys conducted with young people, parents, and other caregivers to identify the design, content, functioning, and user experience of a web-based mental health services navigation tool for children and young people aged up to 25 years in the Australian Capital Territory (ACT). This study was conducted in partnership with the ACT Office for Mental Health and Wellbeing and provides unique insights into the navigation needs of this population, with a dearth of previous research in this area, and the importance of the consultation process in producing an interactive website that is relevant, engaging, and likely to meet the needs of end users.

## Methods

### Participants

A total of 4 online focus groups (N=28), with 5-10 participants each, were conducted. Of these focus groups, 2 focus groups (15/28, 54%; 10/15, 66% female) were conducted with young people aged 16 years and older. These groups were conducted with members of 2 existing youth mental health reference groups: the ACT Youth Advisory Council and the ACT *headspace* Youth Advisory Group, who had experience representing the wider interests and views of their peers. The other 2 focus groups involved parents or caregivers (13/28, 46%; 12/13, 92% female). Participants for these were drawn from expressions of interest from the community and included parents with and without mental health service experience. To attract diverse representation in the focus groups, we advertised for recruitment through ACT mental health sector newsletters and on the social media website Facebook. Facebook advertising targeted adults in the ACT who identified as a parent or caregiver of a child aged 10-25 years. The online modality for the focus groups was chosen to maximize ease of attendance and minimize potential COVID-19 exposure.

Alongside the focus groups, 150 people participated in an online survey to further garner the perspectives of young people and parents or caregivers. Participants were young people aged 16-25 years (53/150, 35%; 44/53, 83% female; mean age 20, SD 2.8 y) and the parents or caregivers (97/150, 65%; 90/97, 93% female; mean age 45.5, SD 7.6 y) of young people aged 0-25 years (0-6 y: 20/97, 21%; 7-11 y: 38/97, 39%; 12-15 y: 47/97, 48%; 16-18 y: 30/97, 31%; and 19-25 y: 37/97, 38%). Participants for the survey were recruited through Facebook advertising. The Facebook advertisements were delivered to 21,600 accounts, with 1300 people engaging with the Facebook post and 374 clicking the link and commencing the survey. Of those who commenced the survey, 40.1% (150/374) went on to complete and submit the survey for analysis. The majority of participants reported English as their first language (47/53, 89% young people and 93/97, 96% parents or caregivers) and had accessed pediatric mental health services in the past (45/53, 85% young people and 86/97, 89% parents or caregivers). Just over half (28/53, 53%) of the young people who completed the survey identified as lesbian, gay, bisexual, transgender, intersex, queer (LGBTIQ+), whereas 21% (11/53) reported having a disability.

### Ethical Considerations

Ethical approval for this study was obtained from the Australian National University Human Research Ethics Committee (protocol 2020/200). All participants were provided with an information sheet outlining the study. Participants in the focus groups were required to return a signed consent form via email before attending the online focus group, whereas survey participants consented to participate in the survey by checking a box at the beginning of the survey. All survey data were collected anonymously, whereas all focus group data were deidentified before analysis and data storage. All focus group participants were provided with a small honorarium of AU $60 (US $40) in recognition of their time and contribution to the study.

### Materials

The focus groups were structured, with a series of guiding questions and prompts that explored the views of young people and parents or caregivers on the potential design, content, functioning, and user experience (eg, optional phone and email support) of a web-based navigation tool (the full list of questions and prompts is given in Multimedia Appendix 1). Questions and prompts were developed in collaboration with staff members from the ACT Office for Mental Health and Wellbeing to gather information required to progress website development from an early concept and include youth and parent perspectives in the design process. Participants were encouraged to share their perspectives verbally with the group or to note any thoughts or ideas in the chat function.

The online survey assessed a range of topics relevant to the development of a youth navigation tool including website features (eg, information, links, quizzes, and service contact details), features and topics by which services could be searched (eg, cost, location, age, and gender), navigation support (eg, phone and online), and possible account functions (eg, acceptable account details and storage of information). Questions were developed with iterative feedback from the ACT Office for Mental Health and Wellbeing and the Youth Coalition of the ACT (peak body for youth affairs in the ACT) to ensure that they covered key issues for website design and were appropriate and accessible for young people. Participants were asked to share their preferences through a combination of close-ended and free-text responses.

### Procedure

Because of the COVID-19 pandemic, all focus groups were conducted online in June 2020 using the Zoom videoconferencing platform (Zoom Video Communications). Given that this study was conducted in partnership with the ACT Office for Mental Health and Wellbeing, all focus groups were cofacilitated by a member of the research team and the ACT Office for Mental Health and Wellbeing. A second member of the research team was also present at all focus group sessions. Their role was to take notes, assist with linking participants with clinical support (if required), and monitor any discussion via the chat function, which was encouraged. All focus groups ran for approximately 90 minutes, online discussions were audio recorded, and any chat conversations were also saved for inclusion in the analysis. One author (ARM) listened to each focus group recording and took detailed point-by-point notes on the content of participant discussions. These were combined with researcher notes taken during the session and participants’ chat conversations for analysis. Because of this approach, all illustrative quotes were drawn from survey data. Participants were provided with help-seeking resources at the conclusion of each focus group, and clinical support or debriefing was available on request.

Two separate surveys were developed for young people and parents or caregivers and were administered online in June 2020 using Qualtrics online survey software (Qualtrics). Surveys took approximately 20 minutes to complete. All participants were provided with a list of help-seeking resources at the conclusion of the survey.

### Data Analysis

Qualitative data were managed using the NVivo 12 software (QSR International). A thematic analysis [17,18] was conducted by 1 author (ARM) on focus group data notes, chat conversations, and free-text survey responses to identify and summarize the key topics and preferences raised by parents and young people within each area of questioning, while preserving the breadth and diversity of perspectives presented. ARM is a lived experience academic with personal experience of mental health service use and professional expertise in youth mental health and service evaluation. Analyses and developing categories were regularly discussed with other members of the research team, including researchers and ACT Office for Mental Health and Wellbeing staff members present at the focus groups, to test assumptions and clarify developing categories. A combination of deductive and inductive approaches to coding was applied. Before commencing data analysis, an initial broad coding framework was developed based on the key areas of questioning during the focus groups: content, features, user experience, and design. Focus group data were deductively coded within each category. As coding progressed, subcategories of describing specific areas of preference and new categories not represented by the deductive framework were developed inductively. A detailed coding framework was developed from the focus group data, including the original broad areas of questioning and inductively developed categories and subcategories. This framework was applied to free-text survey responses, adjusting the framework as necessary to adapt to new information. Finally, data were examined across categories to construct common themes in participants’ preferences. The key topic areas were summarized and grouped under 2 broad categories: the most important *types of information* for a service navigation tool and important *website features*.

## Results

### Overview

The overarching topic areas arising from the focus groups and surveys covered the *types of information* needs identified by young people and parents and the *website features* that would be beneficial to support the mental health of young people. Themes collectively developed from the focus groups and surveys are described within these 2 areas. Data from the focus groups and surveys are integrated throughout this section. Where findings relate only to a specific group (parents or young people) or data collection format (focus group or survey), this is noted in the text.

### Types of Information

#### Up-to-Date Information About Services

Preferences and needs for mental health service information were key topics of discussion in the focus groups and one of the most frequent topics raised in open-ended survey responses. Participants emphasized that it was important for the information on a mental health service navigation website to be regularly updated, with an indication of when updates last occurred, and for no information about listed services to be missing. Content should be relevant to the local region targeted by the website (ACT and surrounding region), as national-level websites can be difficult to navigate and regional content may better cover the full spectrum of services available in that area.

Anything that is Canberra specific is great - wait times, contacts, prices, explaining which professionals do what. For general advice there are lots of national mh [mental health] services already.

Young person, survey participant

It would need to be regularly updated to match service changes. It would need to be all evidence based.

Parent, survey participant

Current and accurate information about service wait times was particularly desirable for all participant groups. Families commonly experienced a lag between identifying that a young person had a problem and being able to access support services. Providing wait time information on a service navigation website could change the way families choose to access services and direct them to services with better availability. Knowing about wait times at the beginning of the help-seeking process would also inform young people and families about likely time frames for receiving support and enable them to plan ahead.

I think the idea of including information about wait list times is very important - as this is usually critical at the time that you are seeking information and support.

Parent, survey participant

I think most people are primarily looking for immediate reassurance and advice. If they can get an idea on how long it might take to access different services this also gives them a time-frame so they can plan.

Parent, survey participant

Young people highlighted the value of a navigation website in providing detailed information about services that would help them make informed choices. Information about accessibility was particularly important for young people. Specifically, cost and parental consent were identified as key barriers to service access, which could be partly addressed by providing detailed cost information (eg, service fees, rebates, and what benefits a child or family may be entitled to), indicating whether parental consent was required to use a service, and noting any relevant legal considerations. Young people also wanted to know if they would be able to physically access a service before they arrived and recommended that the website include information about wheelchair access (eg, presence of ramps and elevators), accessible and gender-neutral toilets, the languages spoken by staff members, whether tailoring was available for people who are vision- or hearing-impaired, and the age range targeted by a service. The content of a service navigation website should also specifically address issues faced by different minority groups, including LGBTIQ+ communities, people with disabilities, and migrant communities.

Along with a list of mental health services, also ways of accessing them, with or without parental involvement and potential cost.

Young person, survey participant

Information specifically geared towards minority groups (BIPOC, LGBTQIA+, people with disabilities etc.). Having information about different mental health issues is great but it would be a mistake not to factor in the relationship between mental health and other aspects of people’s identities.

Young person, survey participant

Parents wanted a navigation website that provided clear information about the kinds of services available in their area, including government and private services and general practitioners who specialize in youth mental health. The ideal website would provide specific information about how to contact services, referral pathways, referral requirements, and whether new referrals were being accepted. Details about service specializations and appointment options (eg, availability of telehealth appointments) were also indicated as helpful across both participant groups.

Information about accessing public v [versus] private mental health services and explaining the difference.

Parent, survey participant

Make sure info on telehealth is clear on if it’s: via the internet text-based, internet video-call, via the telephone, via text message. Don’t just say “this place offers telehealth services.”

Young person, survey participant

#### How to Seek Help, and What Happens Next?

Parents emphasized that a service navigation website should include practical information about how to navigate the whole help-seeking process—from recognizing signs and providing support for their child through to good questions to ask a general practitioner when seeking a mental health referral and how to advocate for and manage the ongoing care of their child. Real stories of how other families navigated mental health issues, practical step-by-step instructions, and diagrams were suggested as useful tools to facilitate access to appropriate services. Young people also wanted clear step-by-step instructions describing how to access help, including how to access services without parental involvement. They noted that finding and visiting a new location can be scary. Providing extra information about the service location, including a map, pictures of the building and the front door, and pictures of staff members, could make the process easier. Information about different types of therapies and how they work were also considered helpful for young people.

Lists/help navigating processes - how to get a mental health plan [Mental Health Treatment Plan], how to see a specialist...who to see for gender dysphoria issues etc.

Parent, survey participant

Maybe some little (short time, a few minutes only) video scenarios with intros to the various services to give a sense of what / who is involved, what to expect...to get a sense of what the service is about...

Parent, survey participant

Participants noted that help-seeking resources should foster hope, rather than disappointment, if a service access attempt did not work out. For example, a parent who completed the survey suggested that a service navigation website could illustrate what a good mental health service arrangement looks like and let people know it is okay to try different clinicians and services if the first referral was not a good fit. Parents also suggested that a navigation website should include easily accessible information on how to recognize, respond to, and seek urgent help during a crisis situation. One parent suggested including a function that could directly link people to a crisis service if needed (eg, a crisis telephone service like Lifeline); however, other participants felt that most people would already know about commonly advertised crisis hotlines.

Information on what a good mental health service arrangement should look like - depending on individual need - and it is ok to not just stick with one person/service forever or choose none.

Parent, survey participant

I think you need information clearly on the homepage about crisis care...and other urgent items that you don't want to have to sift through a website for.

Parent, survey participant

#### Defining Website Scope

In all 4 focus groups and the online surveys, participants raised questions about the ideal scope of a service navigation website. Participants tended to agree that the website’s scope should not be too broad. However, there was uncertainty about whether a navigation website should provide information about mental health in general or only provide information about services. Some participants also expressed a preference for a broader focus on health and well-being, rather than limiting information to mental health services. Young people suggested that broader content could include information about career and employment guidance, coping with current events, mental health at school, and quick references for self-management strategies that could be used while waiting for services (eg, mindfulness, distress tolerance, and coping with panic attacks). Parents were interested in content about mental illnesses (including mood, anxiety, and eating disorders), how to recognize them in specific age groups, and information about common comorbidities and related issues like aggression and self-harm.

This is far too mental illness and mental health service focused than I would be wanting. As a parent it’s helpful to have that information but I would also like resources that are tailor made for the site and have a focus on more on early intervention and mental wellbeing.

Parent, survey participant

Providing links to different websites or existing online programs was suggested as an acceptable option to prevent the scope of a service navigation website from becoming too broad. For example, a navigation website could provide links to existing early intervention and mental well-being resources to support young people’s and parents’ well-being and assist parents to provide support when issues were first identified. Young people suggested providing links to research papers; stories about other people’s lived experience; and different forms of media that represent mental health in a productive way, such as video games, books, and movies. Participants noted that this approach could also connect a website to resources that fall beyond what they would typically define as a “mental health service,” including information about physical health, disability services, drug and alcohol services, and community programs and events that support and empower young people.

I would like to see it link not just mental health services, but other services to help a child overcome all the problems that may be adding to the mental health issue. For example, if the child is experiencing a lot of pain, if the child needs weight management help, if the child is being bullied or needs to develop resilience, if the child is on the autism spectrum, etc.

Parent, survey participant

Stories from people who struggle with their mental well-being but have found support and renewed belief in themselves.

Young person, survey participant

### Website Features

#### Website Design

Participants generally agreed that website design would be an important element of a service navigation website’s success. Elements of website design highlighted by young people included a quality user interface that was easy to use and attractive to the intended audience. Young people described a well-designed website as colorful (but not too colorful or gimmicky), engaging, private, welcoming, local, and informed by what we know about young people and how they think. A level of seriousness in the design was required to ensure that the website was viewed as a reliable resource. Government-branded websites were viewed as a trustworthy source of local information but could be off-putting for young people who had previous negative experiences with government mental health services. A list of organizations that support the website could also signal the trustworthiness of information. Young people were also very aware of accessibility issues, recommending that a navigation website be designed to work across different platforms, for people with slow internet connections, and that it met relevant accessibility standards (eg, for people with low vision or lower literacy levels). A parent suggested that the name of the website was also important and needed to be inviting, explanatory, and nonstigmatizing.

Easy website navigation was another key design issue. When trying to communicate about one’s own mental health in a difficult time, participants felt that the most important thing was to access information easily. Participants indicated that they would be more likely to visit a service navigation website for a specific need or to find specific information. The website content should be set out plainly, with clear pathways to the kind of information the user is looking for. Website design should not be overly complicated, avoiding the need to navigate through tabs and the presence of too many distractions (eg, moving images or videos). Young people were described as multitaskers; thus, a service navigation website needed to capture their attention quickly. To achieve this, it would be helpful to present information clearly and concisely, and to avoid walls of text that could be overwhelming. A frequently asked question section and fact sheets were seen as helpful, but only when presented as a dedicated webpage and not solely as a downloadable document.

#### Search Engines

Parents and young people agreed that a service navigation website should systematically connect people to relevant services quickly. A good quality search engine and filtering system was a particularly important aspect of young people’s user experiences; the search bar can be the first port of call for young people trying to find help. A good search engine was described as easy to navigate, with tags and search terms updated as service information changes. In 1 focus group, young people suggested that a service navigation website could be designed like a nice online shopping experience, with tabs and subtabs for different categories of services and filters that allow users to refine their search and locate the most relevant services. However, 1 parent survey participant noted that a filter system would make them feel terrible if it indicated that there were no services matching their child’s needs, indicating that a balance between detail and generalizability may be required. Participants suggested that if there were no services meeting a young person’s search criteria, the website could direct them to resources, fact sheets, or other information they may find helpful.

...a search engine that finds services that are relevant to you. E.g. you could put in the tags “stressed” “self harming” “aged 17” “don’t want to involve parents” and the search engine would suggest; things you could do to help yourself, services you could access, and a helpline.

Young person, survey participant

The filter would make me feel terrible if meant that my child had no services available so I think that you need to be careful regarding putting too many.

Parent, survey participant

Focus group participants suggested having a quiz or questionnaire to help young people and parents navigate the website. For example, a pop-up box could appear when a person first accessed the website with some questions about what visitors are looking for. Answers could direct young people and parents to appropriate website sections, services, or self-help strategies. Parents suggested that a navigation website could include a symptom checklist, providing recommendations on whether a person needs to seek help from a health professional and within what time frame. Participants noted that any quizzes or checklists should be accompanied by a disclaimer stating that the website could not provide a diagnosis, and questions should be symptom or problem based, not diagnosis based. The results should be anonymous unless a young person chose to disclose them. However, some participants thought it would be important to determine if a young person was in immediate danger and requiring assistance. To facilitate help-seeking, a symptom checklist tool would ideally lead to an outcome, such as connecting users with a real person who can assist with identifying an appropriate service or next step.

Wouldn’t it be easier to complete a mental health survey upon entering the site that directs you to all the relevant pages?

Young person, survey participant

Adolescence is a tricky time. Parents don’t know when to worry, when to escalate to professional help, and when to leave kids to muddle through. A quiz that helps navigate that would be super helpful.

Parent, survey participant

#### Supported Navigation

Participants were enthusiastic about having the option to contact a person, by phone or text-based chat, to help them gauge the seriousness of their issue, navigate the mental health system, connect with appropriate services, and answer questions about what to expect at an appointment. Parents emphasized the importance of creating a sense of trust, confidence, and reliability when a person makes a connection through a navigation website. Finding the right service could take a lot of time, research, and mistakes, particularly during times of stress. Some participants described help seeking as overwhelming, emphasizing the importance of positive experiences that could renew confidence in seeking help and support future service use. An interaction with a real person could be an opportunity to foster hope, positive regard, empathy, reassurance, and a sense of not being alone.

This is a great idea. I found navigating the system to be impossible to start with. I was googling everything, calling all these people, being passed from service to service and getting nowhere. None of the service providers knew what any of the other service providers could do. Someone to help you navigate that would be amazing - especially if you are out of your mind with worry and sleep deprivation like I was!

Parent, survey participant

When asking questions about mental health problems, some young people reported preferring to talk to a health professional, whereas some parents and young people suggested that this would be a good opportunity for peers to support young people and carers and that young adult peers may be better able to connect with younger website users. However, 1 young person noted that talking to a healthy peer may be intimidating for some young people. Across the surveys and focus groups, participants suggested that the person they contacted should be genuine, engaged, supportive, friendly, empathetic, unhurried, unscripted, and well trained, with appropriate counseling skills to support stressed or distressed callers. Their understanding of mental health and local health services should be broader than the understanding provided by the caller’s own experiences.

Having an advisory line (telephone or chat) which is supportive, anonymous if wanted, friendly and unhurried, staffed by a real person who is quietly supportive but knowledgeable would be great to help people navigate the system and get a sense of where they are best placed to use their energies in pursuing or connecting with services.

Parent, survey participant

Participants emphasized that the purpose of any phone or text-based contact options and the roles of the people running them need to be very clear. Contact information should be clearly stated and easy to find, and ideally some contact options would be available outside of normal business hours (eg, evenings, nights, and weekends). Participants had concerns around potential privacy issues, particularly related to data collection and storage, and the need to support people who disclose thoughts of suicide and self-harm. Parents recommended that all interactions end with some kind of closure, for example, actions such as making an appointment for the person with an appropriate service or taking a concrete step that progresses the issue, with timely outcomes.

I’d also be more comfortable in knowing how any conversations via the website were recorded and stored since there is always the chance of personal stuff coming up in conversations, and I wouldn’t want that to be accessible by anyone except those who are directly working to help me.

Young person, survey participant

Further, participants discussed the option of receiving a follow-up call or text, after interacting with a person via a service navigation website. Some participants felt that a follow-up call could be comforting for people who were currently on a waitlist, for example, by providing updates on wait times and identifying alternative sources of support. Survey participants were primarily interested in receiving follow-ups related to service access, for example, checking in to see if services had connected with a young person, how effective the service has been, and if the young person’s needs are being met. Although some participants also wanted the opportunity to give feedback about services, some parents felt oversurveyed by mental health services. Other participants felt that a follow-up would not be helpful in all circumstances. For example, 1 parent felt that receiving a phone call may just be more frustrating if accessing services was not going well. Participants agreed that any follow-up from a service navigation website should be opt-in, the user should have control over how and when they are contacted, young people should decide whether their parents are contacted, and any promises made by a navigation service should be honored (ie, call if you say you will call). Choice and control over contact was seen as particularly important for young people living in high-risk situations, where receiving a phone call or message could potentially be unsafe.

I think having options is good, especially for kids in potentially dangerous situations. And I do think texting is popular with kids.

Young person, survey participant

I think it is also important to see how well people went with actually accessing the services and whether their child's needs were met and what negatives there were.

Parent, survey participant

#### Forums, Reviews, and User Accounts

Website functions that could allow users to share information were met with a mixed reception. The options discussed included forums, service reviews, and user accounts. Forums received the most positive reception, but with important safety considerations for implementation. They were described as a positive tool for young people and parents to connect with peers who have had similar experiences and share coping techniques or to connect with health professionals. However, to be safe and useful, a forum or chat room would require careful moderation. Participants suggested that forums could be provided by a service navigation website itself or the website could instead provide links to external, good quality, moderated mental health social media pages or similar services.

Particularly when I was younger, online resources were huge! Whether that was headspace’s online counselling, or online chat forums with other people. I think those chats were absolutely fantastic, however they definitely needed expert moderation.

Young person, survey participant

I'm a little concerned about forums and/or chat rooms. They would need to be carefully monitored to make sure there are no trolls responding negatively or people using cyber bullying.

Parent, survey participant

User reviews of mental health services were raised as a possibility, but this option had both advantages and disadvantages. Reviews could provide information to help young people choose a suitable service and prepare for their own visit. However, mental health service needs and preferences were seen as highly individual; thus reviews may deter young people from accessing services that would actually suit them. One young person suggested that a government “check mark” (ie, accreditation), indicating that services were legitimate, could be an alternative to reviews, and a parent suggested including a feature where young people and parents could post questions to be answered by a website staff member.

User accounts were the least desirable function of a service navigation website. Young people’s responses to implementing user accounts were overwhelmingly negative, due to concerns around privacy, security, safety, stigma, and limiting access to website features for people without accounts. Young people thought their peers may also associate accounts with costs like subscription fees. A small number of survey participants thought it would be helpful to have a single digital record of the services they had tried and a “wish list” of services they would like to try in the future. However, most parents and young people were uncomfortable with the idea of their mental health information being stored online and believed that this could create a barrier to people using a service navigation website. Young people emphasized the need to be able to access a service navigation website anonymously, particularly when living in high-risk situations where internet use was monitored, and some felt that an optional user account would overcomplicate a navigation website. Young people suggested a range of other, less invasive, ways to tailor user experiences, including features that made it easy to save pages to favorites in a browser, copy content to the clipboard, email a link to service contact information, and save records of service contact information into another application (eg, into the Notes or Photos app on a phone).

A record of interactions makes me feel insecure, because I know the website is keeping data on me. This is not just “not helpful,” but distinctly unhelpful.

Young person, survey participant

I would be concerned about a portal where you sign in. While it might be good to have information all in one place, really mental health information belongs in a doctor’s office. If your child is displaying mental health problems, you don’t really want that recorded when you don’t know how that information will be used into the future.

Parent, survey participant

## Discussion

We conducted focus groups and online surveys to inform the design of a mental health services navigation website. Although the research activity was conducted with a specific region in mind, the findings may assist other organizations designing websites or apps to assist young people and parents in navigating mental health systems. Many of the themes represent common issues faced by young people in need of mental health support.

Participants emphasized the need for, and importance of, up-to-date and accurate information about local mental health services (public and private) and guidance on how to access them (including referral pathways). Participants were aware of existing lists and directories of services, but these were described as incomplete, out of date, and difficult to search. Navigation tools were seen to be most helpful if they could provide local, tailored information, including service information that could not be accessed elsewhere. Transparent information about service cost, wait times, and how to access services without parental permission was in particularly high demand. This information could support young people and parents to make informed choices about which services to select and pursue. Future research exploring the specific service information that is needed to adequately meet end-user needs and that translates into actual service contacts would be beneficial.

Similarly to previous research, participants also emphasized the need for information about referral pathways and how to navigate the mental health system [9,19]. Parents and caregivers shared that they often acted like case managers and advocates for their children, and they wanted access to information and resources that could support them. Step-by-step instructions, flow charts, and real-life stories of accessing mental health care, tailored to the local system, could all support young people and their parents on their journeys from first seeking help to accessing specialist services [19].

An effective search engine was an important aspect of the navigation tool’s design. All information within the navigation tool needs to be searchable, and users should be able to refine their search results using relevant filters. Young people and parents felt that they should be able to independently find service information more easily than they could with a web search engine and that a navigation tool should be broadly accessible, functional, easy to navigate, and tailored to its audience in content and appearance. This finding highlights again the importance of the co-design process, and end-user testing, to ensure that the tool developed is fit for purpose and fully meets the needs of those who will use it [15]. Relatedly, digital tools should also be designed in accordance with web accessibility standards to ensure that all users can effectively use them, including those with disabilities [20].

Participants also felt that it would also be helpful for the navigation tool to provide a phone, text, and/or web chat service. Specifically, participants wanted a phone line or web chat option that could help people to identify relevant and appropriate mental health services and support them to decide which service to contact first. Including this feature would reduce the burden on young people and parents to identify, research, and select services alone. Offering an option to receive a follow-up call or text from a phone line or web chat may also be beneficial.

Underlying many of these findings was the need to have control over the help-seeking process. Control over how and when to interact with content is an important concern for young people in the design of online mental health interventions [16,21]. Providing choices was important to participants in our study; both choice in how they could contact a support person through the navigation tool (eg, phone call, web chat, SMS text messaging, or email) and choice in if, how, and when they were contacted by the navigation tool or engaged with a service.

Other potential navigation tool elements, such as being able to create a user account, were not as desirable. Storing records of health information online, particularly mental health information, raised serious concerns around privacy. Generally, participants felt that people would primarily use the navigation tool anonymously and independently. This aligns with previous findings from the development of mental health interventions for young people and adults, which highlighted confidentiality, privacy, and trust in the organization delivering the service as key areas of importance for end users [21-23]. Some people felt that it could be helpful to be able to record their activity (eg, service wish lists) while using the navigation tool and/or service use in a single location, but participants emphasized that user account features should be opt-in. Such features may be off-putting for potential users.

The involvement of end users in the design of the navigation tool was highly valued by the commissioners of this research and resulted in the development of a youth navigation website called MindMap [24], which captures the key elements identified by participants. The findings of this study provided formative information for the development of MindMap, although there was additional development and testing involved to create the final website, which is beyond the scope of this paper. MindMap is an accessible web-based tool that provides a comprehensive searchable database of local services and provides a clear description of the service, its location, and potential wait times. It is an initiative of the ACT Office of Mental Health and Wellbeing and delivered by a nongovernmental organization with strong connections into the local mental health sector, enabling frequent refreshing of service information. Users of MindMap can use the navigation tool independently or receive navigation support during the week or on weekends from members of the MindMap team via telephone, email, or web chat. Young people and parents or caregivers were involved in the iterative development and testing of MindMap to ensure that it continued to meet their needs.

There are some limitations to this research that should also be considered. First, the participants in the focus groups and survey may not have been representative of all young people and parents or caregivers in the community, and the study may have attracted people with a greater interest in mental health. Preferences for website appearance, content, and features may have varied by age and personal experience of mental health issues [16,25]. Future research would benefit from sampling more young men, and young people and parent or caregivers without mental health service experience, to ensure that the needs and preferences of all targets are adequately captured and met.

The scope of the questions in the focus groups may also have guided the discussion, placing more emphasis on the areas covered by the questions and consequently may have missed other issues. This issue was partly mitigated by providing time within focus groups for participants to identify issues not covered by the questions. The timeline of the project necessitated the use of point-by-point note-taking from focus group recordings, rather than verbatim transcripts, for data analysis. We acknowledge that this approach may have a higher risk of introducing research bias; however, this was mitigated through regular discussion of the analysis with the research team and the inclusion of qualitative survey data in the analysis. Lastly, the research was conducted with a specific region in mind, and thus not all findings may be relevant to other contexts.

Overall, this study provides important insights into the navigation needs of young people and their parents or caregivers seeking mental health services and how best to support them in this process. The focus groups and surveys identified the need for tailored local information, the provision of up-to-date service details, and the opportunity for users to navigate the site independently or with support. Ensuring that young people and their parents or caregivers can access mental health services in an efficient and timely manner is essential to the longer-term health and well-being of young people. Future research assessing the effectiveness of navigation tools in meeting this goal should be strongly encouraged.

## Appendix

Multimedia Appendix 1 Focus group questions and prompts.

## Abbreviations

ACT: Australian Capital Territory
LGBTIQ+: lesbian, gay, bisexual, transgender, intersex, queer

## Data Availability

The data sets generated and analyzed during this study are available from the corresponding author on reasonable request.
