# Supplementary material for: Designing a Web-Based Navigation Tool to Support Access to Youth Mental Health Services: Qualitative Study
Source: JMIR Form Res. 2024 Jan 18;8:e48945. doi: 10.2196/48945 (PMC10835581; doi:10.2196/48945)
Supplement: Multimedia Appendix 1 [file formative_v8i1e48945_app1.docx]

**Multimedia Appendix 1**

**Focus group questions and prompts**

**Q1A: What do you think are the main features/elements of a website that a young person in the ACT would need to support their mental health?**

Prompts: Is there anything you would particularly want? Anything that would put you off? Think about mental health websites, or similar websites you have used in the past: What makes a good website? What makes a website hard to use?

**Q1B. Imagine that you need help to understand the information on the website or that you want some help to try and find support for your mental health and wellbeing. What would be the best/easiest ways for the website to connect you to a person who could help?**

Prompts: Health professional or someone else? Live text chat, voice/video chat, phone, call-back service, email.

**Q1C: After using the website, or getting assistance to locate a service, would you be interested in receiving a follow-up from the website? Why or why not? How would you like the follow-up to occur?**

Prompts: Online survey, personalised email, phone call, text message- is there another way?

**Q1D: [show diagram] Here is an initial design for the website. Are there any parts of this design that you think should/shouldn’t be included?**

Prompts: How helpful do you think each part would be? Are there any parts that would put you off? If you were having problems, e.g. with anxiety, which sections do you think you would use? Why? Is there anything missing?

**Q2: Would you prefer the website to be available within ACT Health (i.e., from health.act.gov.au) or separately?**

Prompts: Are government hosted websites more or less trustworthy? In your experience, are they harder or easier to navigate than separate ones?

**Q3: Is there anything else you would like to suggest?**

Prompts: Are there any ideas for design that we haven’t covered? Do you have any concerns about the website that we haven’t talked about yet?
